# Influence of oxygen concentration on the neuroprotective effect of hydrogen inhalation in a rat model of cardiac arrest

**DOI:** 10.3389/fneur.2022.996112

**Published:** 2022-09-29

**Authors:** Jianjie Wang, Yiming Shen, Jingru Li, Bihua Chen, Changlin Yin, Yongqin Li

**Affiliations:** ^1^Department of Biomedical Engineering and Imaging Medicine, Army Medical University, Chongqing, China; ^2^Department of Emergency, Chongqing Emergency Medical Center, Chongqing, China; ^3^Department of Intensive Care, Southwest Hospital, Army Medical University, Chongqing, China

**Keywords:** cardiac arrest and brain injuries, hydrogen inhalation, neurological outcome, oxygen concentration, targeted temperature management

## Abstract

**Background:**

Post-cardiac arrest (CA) brain injury is the main cause of death in patients resuscitated from CA. Previous studies demonstrated that hydrogen inhalation mitigates post-CA brain injury. However, factors affecting the efficacy of hydrogen remain unknown. In the present study, we investigated the influence of oxygen concentration and targeted temperature on neuroprotective effect in a CA rat model of ventricular fibrillation (VF).

**Methods:**

Cardiopulmonary resuscitation (CPR) was initiated after 7 min of untreated VF in adult male Sprague–Dawley rats. Immediately following successful resuscitation, animals were randomized to be ventilated with 21% oxygen and 79% nitrogen (21%O_2_); 2% hydrogen, 21% oxygen, and 77% nitrogen (2%H_2_ + 21%O_2_); 2% hydrogen, 50% oxygen, and 48% nitrogen (2%H_2_ + 50%O_2_); or 2% hydrogen and 98% oxygen (2%H_2_ + 98%O_2_) for 3 h. For each group, the target temperature was 37.5°C for half of the animals and 35.0°C for the other half.

**Results:**

No statistical differences in baseline measurements and CPR characteristics were observed among groups. For animals with normothermia, 2%H_2_ + 50%O_2_ (123 [369] vs. 500 [393], *p* = 0.041) and 2%H_2_ + 98%O_2_ (73 [66] vs. 500 [393], *p* = 0.002) groups had significantly lower neurological deficit scores (NDSs) at 96 h and significantly higher survival (75.0 vs. 37.5%, *p* = 0.033 and 81.3 vs. 37.5%, *p* = 0.012) than 21%O_2_ group. For animals with hypothermia, no statistical difference in NDS among groups but 2%H_2_ + 98%O_2_ has significantly higher survival than the 21%O_2_ group (93.8 vs. 56.3%, *p* = 0.014).

**Conclusion:**

In this CA rat model, inhaling 2% hydrogen combined with a high concentration of oxygen improved 96-h survival, either under normothermia or under hypothermia.

## Introduction

Cardiac arrest (CA) is a major public health problem with a higher frequency especially in the young (1). Despite decades of research and investment in the field of cardiopulmonary resuscitation (CPR), survival rates after CA remain poor even after achieving the return of spontaneous circulation (ROSC), and up to 70% of patients die from complications (2). Post-CA brain injury, which is encompassed by primary (ischemic) and secondary (reperfusion) injury occurring sequentially during CA, resuscitation, and the acute post-resuscitation phase, is the main cause of death (3). Since the ultimate goal of resuscitation is to restore cardiac and cerebral functions to that before the arrest, post-resuscitation care has been focused on minimizing secondary brain injury and optimizing the chances of recovery following ROSC (4).

Among all post-resuscitation interventions suggested and/or recommended, targeted temperature management (TTM) or therapeutic hypothermia is the most persuasive treatment that has proven benefits both for the neurological recovery and survival (5). According to the 2015 International Resuscitation Guidelines, the targets of temperature and duration are between 32 and 36°C for at least 24 h for all cardiac rhythms in adult patients with CA (6). In spite of that, TTM has been extensively studied, its neuroprotective effect remains controversial. Unfortunately, recent clinical studies fail to show beneficial effects between TTM and normothermia on neurologic recovery and mortality outcomes (7, 8). Therefore, treatment of post-CA brain injury remains a big challenge.

Since the discovery that molecular hydrogen protects the brain against ischemia-reperfusion injury by selectively neutralizing reactive oxygen species (ROS), the neuroprotective effects of hydrogen have been studied in variety of animal models (9). These studies have consistently demonstrated that hydrogen greatly mitigates post-CA brain injury and improves neurological outcomes, to an extent comparable or superior to TTM in different CA models (10–20). As hydrogen has been shown to be well tolerated for clinical use in healthy adults and to be a feasible therapy for patients with post-CA brain injury, a multi-center randomized trial is ongoing to confirm the efficacy of hydrogen on neurological outcomes in comatose out-of-hospital CA survivors (21–23).

Hydrogen inhalation is the most commonly used way for hydrogen administration in both animal and clinical studies since mechanical ventilation is routinely utilized in the post-resuscitation period. In fact, hydrogen needs to be mixed with a certain concentration of oxygen at a certain temperature during mechanical ventilation. The optimal and safe concentration of hydrogen is demonstrated to be 2%, but the optimal oxygen concentration and targeted temperature for hydrogen inhalation are undetermined (9, 10). The objective of this study was designed to investigate the influence of oxygen concentration and TTM on the neuroprotective effect of hydrogen in a CA model of ventricular fibrillation (VF).

## Materials and methods

This prospective, randomized, observational animal study was approved by the Laboratory Animal Welfare and Ethics Committee of the Army Medical University (protocol number: SGX2018yjs02). One hundred and seventy-eight male Sprague–Dawley rats, weighed between 270 and 361 g (10–12 weeks of age) and supplied by a single-source breeder (Laboratory Animal Center, Army Medical University, Chongqing, China), were used for this study. The sample size was determined by our previous animal studies that investigated the effects of hydrogen, oxygen, and TTM (14, 16, 18, 24). The study was in accordance with the Animal Research: Reporting of *In Vivo* Experiments (ARRIVE) guidelines and all animals received humane care in compliance with the Principles of Laboratory Animal Care and Guide for the Care and Use of Laboratory Animals.

### Animal preparation

After fasting overnight, the animals were anesthetized with an intraperitoneal injection of pentobarbital (45 mg/kg). Additional doses (10 mg/mg) were administered intravenously at ~1-h intervals to maintain anesthesia. The tracheas of the animals were intubated through a tracheotomy with a 14-gauge cannula and mechanically ventilated with a tidal volume of 6.5 ml/kg at an FiO_2_ of 0.21 (R415, RWD Life Science Co. LTD, Shenzhen, China). A PE-50 catheter was advanced from the right femoral artery for measurement of arterial pressure and blood sampling. The left femoral vein was cannulated with a PE-50 catheter to administrate fluids and drugs. Arterial pressure and lead II electrocardiogram (ECG) were continuously measured by a multi-parameter monitor (Model 90369, Spacelabs, Snoqualmie, WA, USA). Core temperature was monitored by a thermocouple probe (TH-212, Bjhocy Science and Technology Co. Ltd., Beijing, China) that was placed into the esophagus and maintained by a heating lamp to ensure appropriate temperature management. All catheters were flushed intermittently with saline solution containing 2.5 IU/ml heparin.

### Experimental procedures

Mechanical ventilation was discontinued, and VF was induced by 50 Hz transesophageal cardiac pacing. CPR was begun after 7 min of untreated CA. Manual external chest compression was performed at a rate of 240/min by the same investigator. Coincident with the initiation of chest compression, animals were mechanically ventilated at a frequency of 90/min with a tidal volume of 6.0 ml/kg and an FiO_2_ of 1.0. A dose of epinephrine (0.02 mg/kg) was injected 1 min after the start of CPR. A defibrillation shock was attempted with a 2-J biphasic waveform (M Series, Zoll Medical Corporation, Chelmsford, MA, USA) after 3 min of CPR. Chest compression was immediately resumed after the shock delivery until the confirmation of ROSC. ROSC was defined as the return of an organized cardiac rhythm with a mean arterial pressure (MAP) >60 mmHg for an interval >5 min.

Immediately following ROSC, animals were randomized to 4 groups (*n* = 32 each) and mechanically ventilated with 21% oxygen and 79% nitrogen (21%O_2_, also served as control); 2% hydrogen, 21% oxygen, and 77% nitrogen (2%H_2_ + 21%O_2_); 2% hydrogen, 50% oxygen, and 48% nitrogen (2%H_2_ + 50%O_2_); or 2% hydrogen and 98% oxygen (2%H_2_ + 98%O_2_) for 3 h. TTM was administrated for all of the animals, and the target temperature was 37.5°C (normothermia) for half of the animals (*n* = 16) and 35.0°C (hypothermia) for the other half (*n* = 16) in each group. For animals subjected to hypothermia, surface cooling was initiated immediately after ROSC with the aid of ice packs and an electrical fan. Once the target temperature reached 35.0°C, it was maintained for 3 h and gradually rewarmed to 37.5°C over a period of 2 h. We chose 35.0°C because hypothermia is usually defined as a body core temperature below 35.0°C, and a previous study demonstrated that there was no significant difference in survival, neurologic function, or adverse events between patients treated with a target temperature of 33.0 or 36.0°C (25). For animals assigned to normothermia, core temperature was maintained at 37.5 ± 0.3°C until the end of the experiment. Additionally, 10 animals underwent the same preparation operation, but without the induction of VF, the procedure of resuscitation and post-resuscitation care were served as Sham group.

All rats received the same post-operative care, except for the composition and concentration of the inhaled gas and the target temperature. All catheters, including endotracheal tube, were removed and wounds were surgically sutured 6 h after resuscitation. Animals were then returned to their cages with a room temperature maintained at 21–25°C with 12 h of light/12 h of dark exposure and were observed for 96 h.

### Measurements

The primary outcome was 96-h survival and the secondary outcome was neurological recovery. ECG, electroencephalogram (EEG), and blood pressure were continuously recorded with a PC-based data acquisition system (DATAQ Instruments Inc., Akron, OH, USA). Arterial blood samples were drawn at baseline and 3 and 6 h after ROSC. Blood gases were measured with the aid of a blood analyzer (i-STAT, Abbott Point of Care Inc, Abbott Park, IL, USA). Cardiac function was non-invasively measured with an echocardiograph system (DC-6, Mindray Medical International Limited, Shenzhen, China). Left ventricular ejection fraction (LVEF) served as quantitative measurement of myocardial contractile function. The characteristics of the earlier post-resuscitation EEG, including the onset time of the EEG burst (OTOB), time to normal EEG trace (TTNT), and weighted-permutation entropy (WPE), were quantitatively analyzed as described in our previous studies (18, 24).

Neurological deficit score (NDS) was examined and confirmed by 2 investigators blinded to the treatment every 24 h after ROSC. Consciousness, respiration, cornea reflex, cranial reflex, auditory reflex, motor sensory function, and behavior were scored according to an NDS system (0–500 scale; 0 means no observed neurological deficit and 500 represents death or brain death) that was developed to evaluate neurological outcomes after global cerebral ischemia in rats (26). Consciousness was measured by checking spontaneous attention to the environment and reaction to pinching of the ear or tail (0–100 scale). Respiration was reflected by breathing frequency (0–100 scale). Cornea reflex (0–40 scale), cranial reflex (0–30 scale), and auditory reflex (0–30 scale) were respectively measured by touching the center of the cornea with a hemostat, stimulating with a catheter, and banging metal cop with clamp, and their minimal and maximal values represent absent and brisk reactions. Motor sensory function was measured by the reflex that corrects the orientation of the body when it was taken out of its normal upright position (0–100 scale). Behavior (0–100 scale) was scored by the level of spontaneous or stimulated movements.

After assessment of the 96-h NDS, the survived animals and 10 sham-operated rats were anesthetized with pentobarbital sodium. The brains were removed and immersion fixed in 10% neutral buffered formalin. Organs were embedded in paraffin and sectioned (5 μm) on a microtome. The sections in the CA1 hippocampus region were stained with hematoxylin and eosin (H&E) (Servicebio Technology Co, Ltd, Wuhan, China) for morphological evaluation, with terminal deoxynucleotidyl transferase-mediated deoxyuridine triphosphate nick end labeling (TUNEL) (Roche Applied Science, Basel, Switzerland), 4′,6-diamidino-2-phenylindole (DAPI), and Caspase-3 (Servicebio Technology Co, Ltd, Wuhan, China) for apoptosis detection.

In total, 40 rats (*n* = 5 in each group) underwent the same experimental procedure and were anesthetized 6 h after resuscitation. The hippocampus and cortex were removed respectively for the detection of oxidative stress-related biomarkers and compared with those of 10 sham-operated rats. The brain tissue homogenate was centrifuged at 3,500 rev/min at 4°C for 10 min and then the supernatant was collected. The tissue protein concentration was quantified using a bicinchoninic acid protein assay kit (Nanjing JianCheng Technology Co, Ltd, China). The density of superoxide dismutase (SOD) that reflects the ability to scavenging free radicals, malondialdehyde (MDA) that plays a toxic effect on the cells, and 8-hydroxy-2-deoxy guanosine (8-OHDG) that indicates the oxidative damage of DNA were measured according to manufacturer's instructions (Nanjing JianCheng Technology Co, Ltd, China).

### Statistical analysis

The Kolmogorov-Smirnov test was used to confirm the normality of data distribution. Normally distributed data were expressed as means and standard deviations (SD), whereas non-normally distributed data were presented as median and interquartile ranges. Hemodynamic and blood gas variables were compared by two-way repeated measures analysis of variance (ANOVA) followed by Bonferroni correction for post-hoc comparisons. NDSs were analyzed non-parametrically using the Kruskal–Wallis test and then using the Mann–Whitney *U* test for a two-group comparison. Survival curves were obtained with a Kaplan–Meier analysis and compared among groups with a log-rank test. Mutual independence of variables and differences in proportions were evaluated by means of chi-square statistics. Statistical analyses were performed using SPSS software (Version 22.0, IBM Corp. Armonk, NY, USA). A *p* < 0.05 was regarded as statistically significant.

## Results

There were no statistical differences in baseline physiological measurements and characteristics of CPR among groups, either under normothermia (Table 1) or under hypothermia (Table 2). All animals were included for analysis and no adverse events were observed during the experimental periods.

**Table 1:** Baseline and cardiopulmonary resuscitation (CPR) characteristics for animals under normothermia.

**Characteristics**	**21%O_2_ (*n* = 16)**	**2%H_2_ + 21%O_2_ (*n* = 16)**	**2%H_2_ + 50%O_2_ (*n* = 16)**	**2%H_2_ + 98%O_2_ (*n* = 16)**
Body weight (g)	318.6 ± 24.5	319.5 ± 22.7	316.8 ± 22.3	315.4 ± 19.1
Heart rate (BPM)	414.8 ± 30.0	391.4 ± 46.0	418.1 ± 30.5	404.8 ± 25.7
MAP (mmHg)	126.1 ± 7.6	121.1 ± 8.4	125.3 ± 6.7	123.1 ± 10.1
Temperature (°C)	37.5 ± 0.2	37.5 ± 0.2	37.5 ± 0.2	37.5 ± 0.1
PETCO_2_ (mmHg)	37.6 ± 4.9	37.4 ± 2.8	37.1 ± 6.0	38.1 ± 4.7
LVEF (%)	83.0 ± 1.0	82.2 ± 2.1	83.3 ± 1.7	83.2 ± 2.8
CPR duration (s)	215.5 ± 24.0	210.5 ± 22.7	209.7 ± 20.2	205.3 ± 26.2
Defibrillations (*n*)	2.1 ± 0.8	1.9 ± 0.9	1.8 ± 0.8	1.8 ± 0.9
Epinephrine (μg)	17.5 ± 6.9	17.8 ± 5.5	16.9 ± 5.7	17.2 ± 5.3
Pentobarbital (mL)	1.3 ± 0.2	1.3 ± 0.3	1.3 ± 0.2	1.4 ± 0.2

BPM, beats per minute; MAP, mean arterial pressure; PETCO_2_, partial pressure of end-tidal carbon dioxide; LVEF, left ventricular ejection fraction.

**Table 2:** Baseline and cardiopulmonary resuscitation (CPR) characteristics for animals under hypothermia.

**Characteristics**	**21%O_2_ (*n* = 16)**	**2%H_2_ + 21%O_2_ (*n* = 16)**	**2%H_2_ + 50%O_2_ (*n* = 16)**	**2%H_2_ + 98%O_2_ (*n* = 16)**
Body weight (g)	313.5 ± 19.7	316.9 ± 22.8	313.4 ± 20.1	317.2 ± 19.7
Heart rate (BPM)	412.7 ± 25.9	405.2 ± 31.7	403.0 ± 39.5	402.7 ± 39.7
MAP (mmHg)	125.9 ± 8.5	120.9 ± 8.1	123.9 ± 8.4	126.0 ± 6.1
Temperature (°C)	37.5 ± 0.2	37.5 ± 0.2	37.4 ± 0.2	37.5 ± 0.2
PETCO_2_ (mmHg)	37.5 ± 5.7	38.0 ± 2.9	37.1 ± 3.7	37.2 ± 3.3
LVEF (%)	82.7 ± 1.0	83.9 ± 3.0	83.6 ± 1.9	82.8 ± 2.2
CPR duration (s)	215.7 ± 27.0	215.1 ± 46.7	212.1 ± 23.8	209.1 ± 32.0
Defibrillations (n)	2.2 ± 1.1	2.1 ± 1.8	1.8 ± 0.7	1.8 ± 1.4
Epinephrine (μg)	16.3 ± 5.9	16.8 ± 7.2	16.3 ± 5.0	16.9 ± 7.5
Pentobarbital (mL)	1.4 ± 0.3	1.4 ± 0.4	1.5 ± 0.4	1.4 ± 0.3

BPM, beats per minute; MAP, mean arterial pressure; PETCO_2_, partial pressure of end-tidal carbon dioxide; LVEF, left ventricular ejection fraction.

### Influence of oxygen concentration on the neurological outcome under normothermia

**Figure 1:**
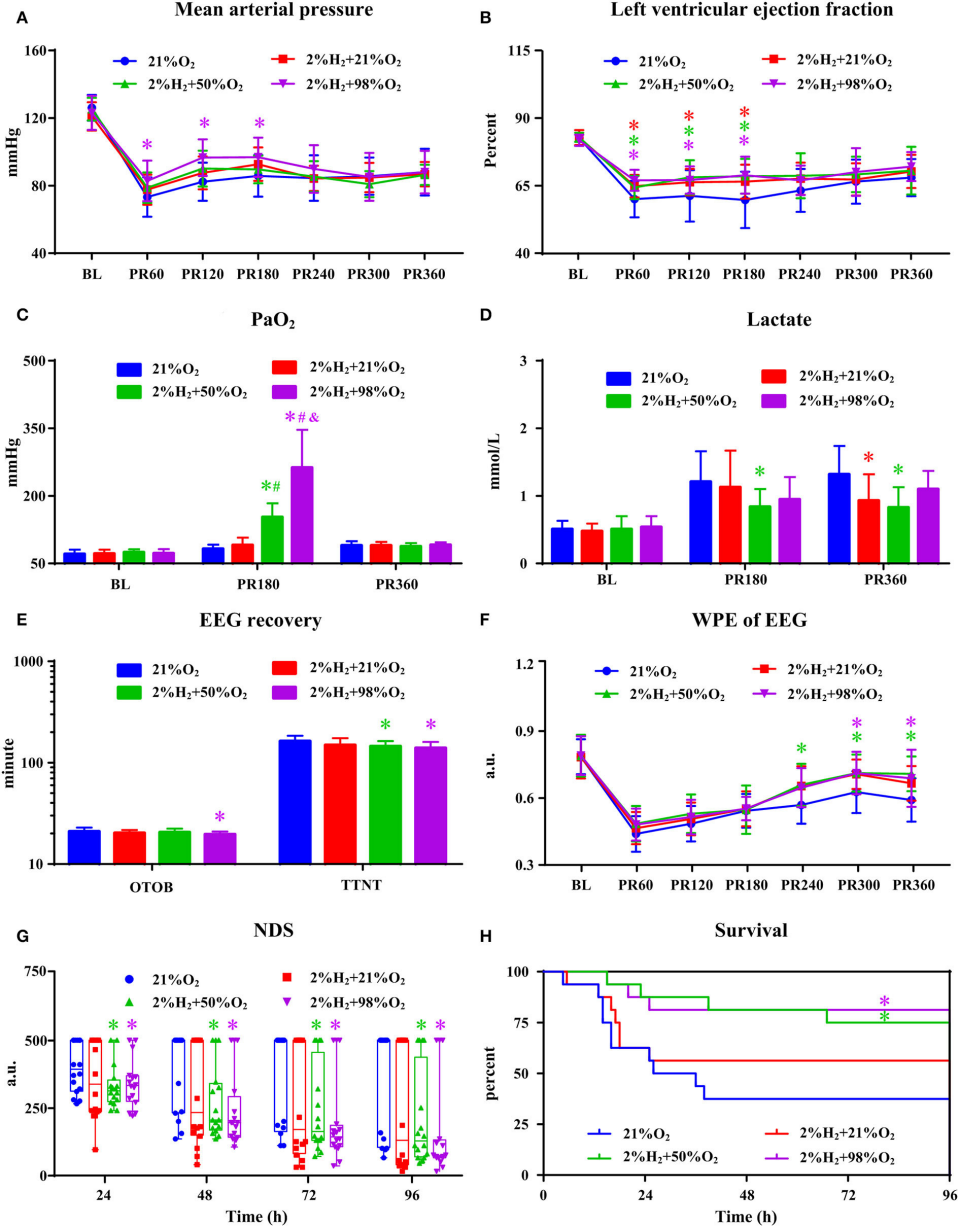
The hemodynamic and neurological data in animals under normothermia. **(A)** Mean arterial pressure (MAP), **(B)** left ventricular ejection fraction (LVEF), **(C)** PaO_2_, **(D)** lactate, **(E)** EEG recovery, **(F)** weighted-permutation entropy (WPE) of EEG, **(G)** neurological deficit score (NDS), and **(H)** survival curve. BL, baseline; PR, post-resuscitation. *p < 0.05 vs. 21%O_2_ group; ^#^*p* < 0.05 vs. 2%H_2_ + 21%O_2_ group; ^&^*p* < 0.05 vs. 2%H_2_ + 50%O_2_ group.

All animals survived the 6 h monitoring period except 1 rat died at 5.5 h in the 2%H_2_ + 21%O_2_ group. The hemodynamic and neurological data are shown in Figure 1. Compared with control, significantly higher MAP in the 2%H_2_ + 98%O_2_ group and LVEF in the 2%H_2_ + 21%O_2_, 2%H_2_ + 50%O_2_, and 2%H_2_ + 98%O_2_ groups were observed during hydrogen halation. PaO_2_ levels were significantly higher in the 2%H_2_ + 50%O_2_ and 2%H_2_ + 98%O_2_ groups. Meanwhile, lactate levels measured in the 2%H_2_ + 21%O_2_ and 2%H_2_ + 50%O_2_ groups were considerably lower. Compared with control, OTOB was significantly lower in the 2%H_2_ + 98%O_2_ group, WPE was significantly higher while TTNT and NDS were significantly lower in the 2%H_2_ + 50%O_2_ and 2%H_2_ + 98%O_2_ groups. In total, 6, 9, 12, and 13 animals in control, 2%H_2_ + 21%O_2_, 2%H_2_ + 50%O_2_, and 2%H_2_ + 98%O_2_ groups survived to 96 h. The cumulative 96-h survival was significantly higher in the 2%H_2_ + 50%O_2_ (75.0 vs. 37.5%, log-rank *p* = 0.019) and 2%H_2_ + 98%O_2_ (81.3 vs. 37.5%, long-rank *p* = 0.013) groups.

**Figure 2:**
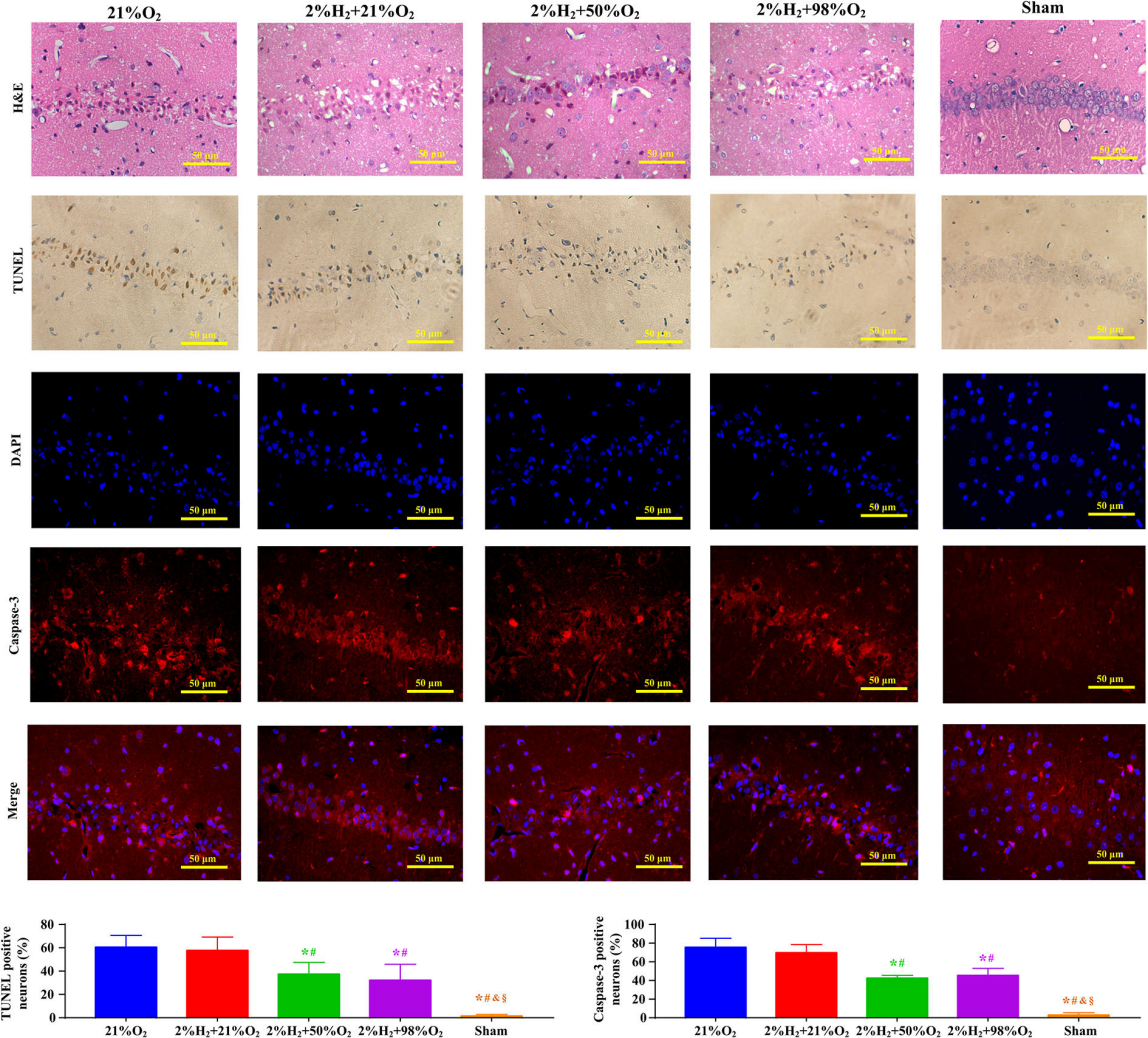
Representative micrographs of the hematoxylin and eosin (H&E), terminal deoxynucleotidyl transferase-mediated deoxyuridine triphosphate nick end labeling (TUNEL), 4′,6-diamidino-2-phenylindole (DAPI), Caspase-3 stained neurons in the CA1 hippocampus region, ratio of TUNEL positive neurons, and ratio of Caspase-3 positive neurons at 96 h after resuscitation in animals under normothermia. *p < 0.05 vs. 21%O_2_ group; ^#^*p* < 0.05 vs. 2%H_2_ + 21%O_2_ group; ^&^*p* < 0.05 vs. 2%H_2_ + 50%O_2_ group; ^§^*p* < 0.05 vs. 2%H_2_ + 98%O_2_ group.

Representative micrographs of the pathological examination are presented in Figure 2. Most neurons in the CA1 region exhibited irregular, polygonal, and spindle shapes, and apoptotic cells were frequently observed in the control group. However, the proportions of abnormal neurons were obviously decreased and relatively fewer apoptotic neurons were observed in the 2%H_2_ + 50%O_2_ and 2%H_2_ + 98%O_2_ groups. At the same time, the ratios of Caspase-3 positive neurons were significantly reduced in the 2%H_2_ + 50%O_2_ (42.4 ± 3.1%) and 2%H_2_ + 98%O_2_ (45.3 ± 7.6%) groups when compared to the control (75.5 ± 9.7%) and 2%H_2_ + 21%O_2_ (69.7 ± 8.7%) groups (*p* < 0.001).

**Figure 3:**
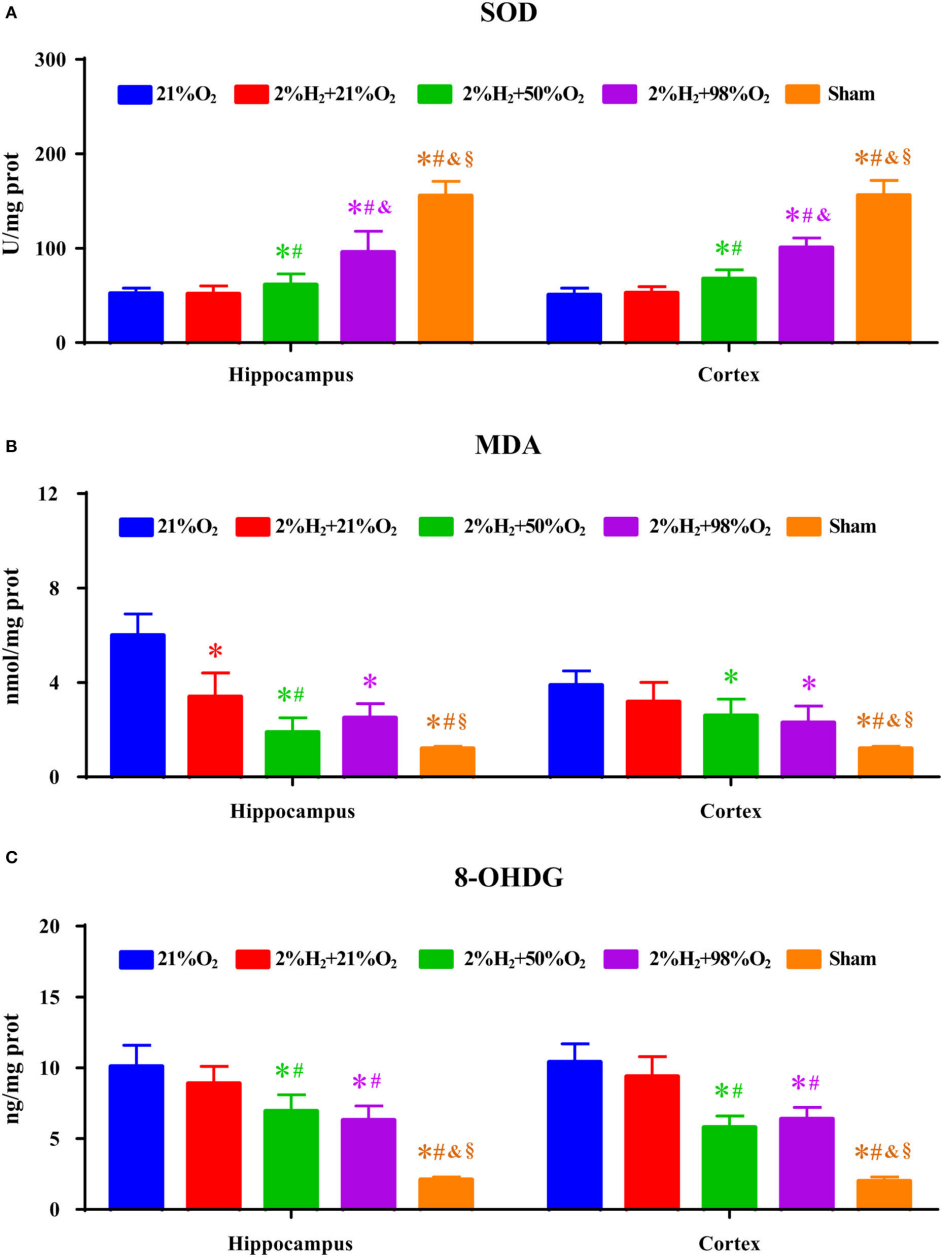
The concentrations of **(A)** superoxide dismutase (SOD), **(B)** malondialdehyde (MDA), and **(C)** 8-hydroxy-2-deoxy guanosine (8-OHDG) of the hippocampus and cortex were measured 6 h after resuscitation in animals under normothermia. *p < 0.05 vs. 21%O_2_ group; ^#^*p* < 0.05 vs. 2%H_2_ + 21%O_2_ group; ^&^*p* < 0.05 vs. 2%H_2_ + 50%O_2_ group; ^§^*p* < 0.05 vs. 2%H_2_ + 98%O_2_ group.

Superoxide dismutase, MDA, and 8-OHDG level measurements of the hippocampus and cortex are shown in Figure 3. Compared with the control and 2%H_2_ + 21%O_2_ groups, the SOD level was significantly increased while the 8-OHDG level was dramatically decreased in the 2%H_2_ + 50%O_2_ and 2%H_2_ + 98%O_2_ groups. Compared with control, MDA level was markedly decreased in all of the 3 hydrogen inhalation groups with the exception of the cortex in the 2%H_2_ + 21%O_2_ group. Additionally, the SOD level of the 2%H_2_ + 98%O_2_ group was considerably higher than that in the 2%H_2_ + 50%O_2_ group.

### Influence of oxygen concentration on neurological outcome with hypothermia

**Figure 4:**
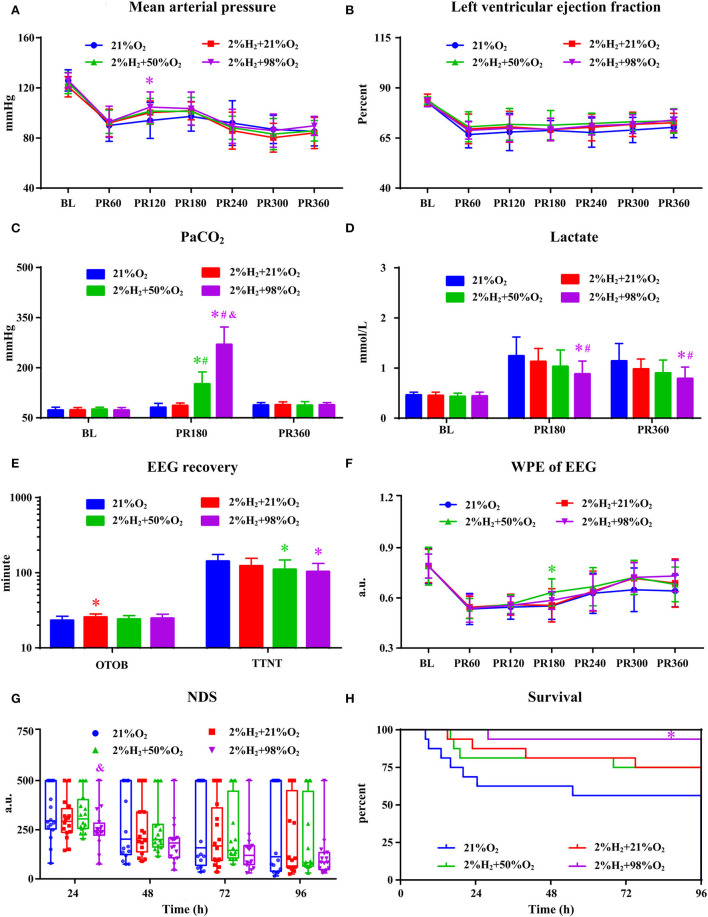
The hemodynamic and neurological data in animals under hypothermia. **(A)** Mean arterial pressure (MAP), **(B)** left ventricular ejection fraction (LVEF), **(C)** PaO_2_, **(D)** lactate, **(E)** EEG recovery, **(F)** weighted-permutation entropy (WPE) of EEG, **(G)** neurological deficit score (NDS), and **(H)** survival curve. BL, baseline; PR, post-resuscitation. *p < 0.05 vs. 21%O_2_ group; ^#^*p* < 0.05 vs. 2%H_2_ + 21%O_2_ group; ^&^*p* < 0.05 vs. 2%H_2_ + 50%O_2_ group.

All animals survived the 6 h monitoring period and no significant differences in hemodynamic measurements among groups except a relatively higher MAP in the 2%H_2_ + 98%O_2_ group. As shown in Figure 4, PaO_2_ levels are significantly higher in the 2%H_2_ + 50%O_2_ and 2%H_2_ + 98%O_2_ groups than in the control and 2%H_2_ + 21%O_2_ groups. Meanwhile, lactate levels were markedly lower in the 2%H_2_ + 98%O_2_ group than in the control and 2%H_2_ + 21%O_2_ groups. OTOB in the 2%H_2_ + 21%O_2_ group was significantly higher, while TTNT in the 2%H_2_ + 50%O_2_ and 2%H_2_ + 98%O_2_ groups was significantly lower than that of the control. No significant differences were found in WPE and NDS among groups with the exception of a higher WPE at 3 h in the 2%H_2_ + 50%O_2_ group than the control and a lower NDS at 24 h in the 2%H_2_ + 98%O_2_ group than 2%H_2_ + 50%O_2_ group. In total, 9, 12, 12, and 15 animals in control, 2%H_2_ + 21%O_2_, 2%H_2_ + 50%O_2_, and 2%H_2_ + 98%O_2_ groups survived to 96 h. The cumulative 96-h survival was significantly higher in the 2%H_2_ + 98%O_2_ group (93.8 vs. 56.3%, *p* = 0.013) when compared to the control.

**Figure 5:**
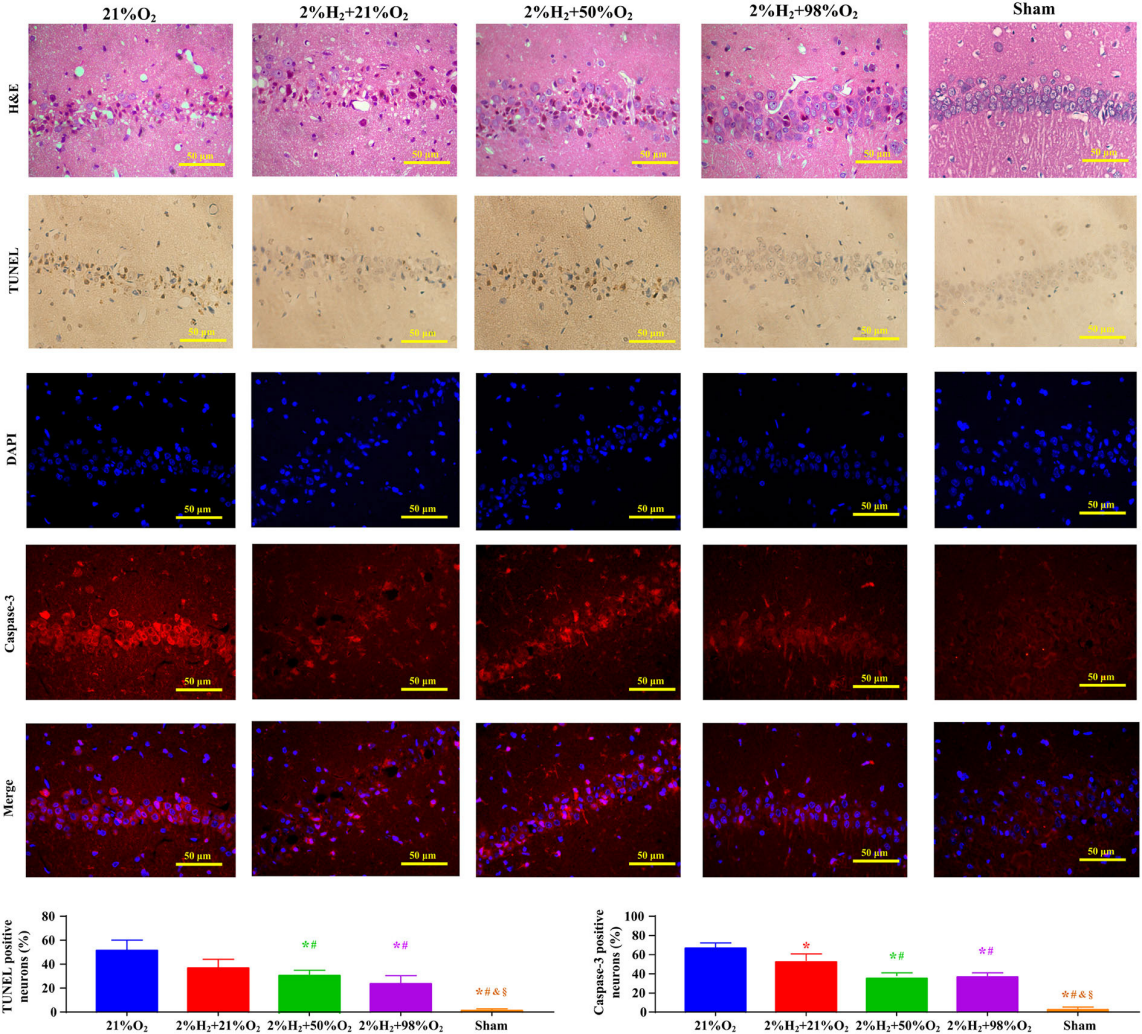
Representative micrographs of the hematoxylin and eosin (H&E), terminal deoxynucleotidyl transferase-mediated deoxyuridine triphosphate nick end labeling (TUNEL), 4′,6-diamidino-2-phenylindole (DAPI), Caspase-3 stained neurons in the CA1 hippocampus region, ratio of TUNEL positive neurons, and ratio of Caspase-3 positive neurons at 96 h after resuscitation in animals under hypothermia. *p < 0.05 vs. 21%O_2_ group; ^#^*p* < 0.05 vs. 2%H_2_ + 21%O_2_ group; ^&^*p* < 0.05 vs. 2%H_2_ + 50%O_2_ group; ^§^*p* < 0.05 vs. 2%H_2_ + 98%O_2_ group.

Representative micrographs of the pathological examination are presented in Figure 5. Compared with control, the proportion of neurons with intact structure was relatively higher and the proportions of degenerated and apoptotic neurons were relatively lower in the 2%H_2_ + 98%O_2_ group. The ratio of Caspase-3 positive neurons was significantly reduced in the 2%H_2_ + 50%O_2_ (35.1 ± 4.6%) and 2%H_2_ + 98%O_2_ (36.3 ± 4.1%) when compared to the 2%H_2_ + 21%O_2_ (52.4 ± 7.9%) and control (64.9 ± 6.0%) groups (*p* < 0.01).

**Figure 6:**
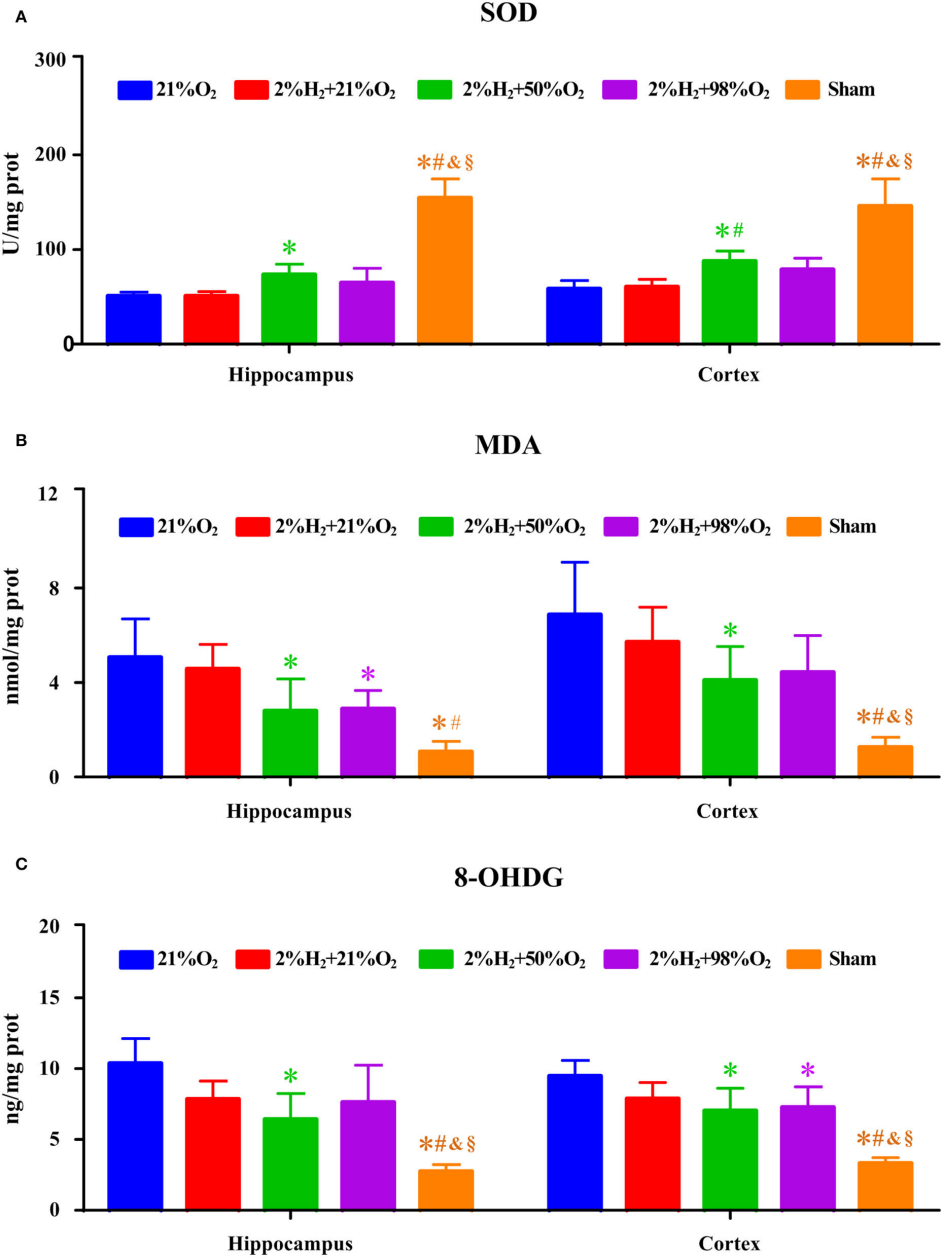
The concentrations of **(A)** superoxide dismutase (SOD), **(B)** malondialdehyde (MDA), and **(C)** 8-hydroxy-2-deoxy guanosine (8-OHDG) of the hippocampus and were cortex measured 6 h after resuscitation in animals under hypothermia. *p < 0.05 vs. 21%O_2_ group; ^#^*p* < 0.05 vs. 2%H_2_ + 21%O_2_ group; ^&^*p* < 0.05 vs. 2%H_2_ + 50%O_2_ group; ^§^*p* < 0.05 vs. 2%H_2_ + 98%O_2_ group. *n* = 5 for each group.

Superoxide dismutase, MDA, and 8-OHDG levels of the hippocampus and cortex are shown in Figure 6. Compared with control, animals in the 2%H_2_ + 50%O_2_ group had significantly higher SOD, lower MDA, and lower 8-OHDG levels in both hippocampus and cortex. At the same time, animals in the 2%H_2_ + 98%O_2_ group had significantly lower MDA levels in hippocampus and significantly lower 8-OHDG levels in the cortex than the control.

### Interaction between hydrogen and oxygen concentration on 96-h survival

Mutual independence analysis revealed that there was no significant association between hydrogen inhalation and temperature management (χ^2^ = 16.3, *df* = 10, $\chi_{0.05}^{2}$ = 18.31, *p* > 0.05). Therefore, hydrogen treatment groups were merged when investigating the effect of TTM. Although the combined 96-h survival rate was relatively higher in animals under hypothermia but did not reach statistical significance when compared with that of normothermia (75.0 vs. 62.5%, *p* = 0.127). Similarly, the normothermic and hypothermic subgroups were merged when investigating the interaction between hydrogen and oxygen concentration. The combined 96-h survival rates were 46.9, 65.6, 75.0, and 87.5% for control, 2%H_2_ + 21%O_2_, 2%H_2_ + 50%O_2_, and 2%H_2_ + 98%O_2_ groups. Compared with control, the survival rates were significantly higher in the 2%H_2_ + 50%O_2_ (*p* = 0.021) and 2%H_2_ + 98%O_2_ (*p* < 0.001) groups. Compared with the 2%H_2_ + 21%O_2_ group, the survival rate was significantly higher in the 2%H_2_ + 98%O_2_ group (*p* = 0.039).

## Discussion

Post-CA brain injury is the common cause of morbidity and mortality. Much evidence has shown that the underlying pathophysiological processes activated within minutes to hours after ischemia and reperfusion injury, including free radical formation, excitotoxicity, disrupted calcium homeostasis, pathological protease cascades, and activation of cell death signaling pathways, contribute to mortality and neurological impairment (27). As one of the neuroprotection methods, TTM can affect many metabolic pathways, reactions of inflammation, apoptosis processes, and promote neuronal integrity. However, the clinical therapeutic effect of hypothermia is limited because its effectiveness is dependent on the basic diseases causing CA and presenting rhythm of the patients (28, 29). At the same time, adverse events and complications of therapeutic hypothermia are common and may inversely be related to neurological recovery (25, 30). Molecular hydrogen has been reported to have anti-oxidant, anti-inflammatory, and anti-apoptotic properties by selectively neutralizing ROS and is currently recognized as an emerging therapeutic approach in cardiovascular and cerebrovascular diseases. Different from hypothermia that decreases cerebral blood flow along with brain metabolism, hydrogen has no known side effects and its use does not disturb cellular metabolic redox reactions, intracellular signaling, or physiological metabolic and enzymatic reactions (31). These properties suggest that hydrogen may be a good candidate for post-CA care as an effective and safe therapy.

In line with previous animal studies, hydrogen inhalation alleviated post-CA brain injury and improved 96-h survival by its anti-oxidant and anti-apoptotic functions in the present study (10, 13, 14, 16, 18, 19). Moreover, our study extended previous observation on the therapeutic mechanism of hydrogen and provided new evidence on confounding factors influencing its efficacy. Although there was a trend to improve the survival after inhaling hydrogen with 21% oxygen, it did not reach statistical significance. This indicated that the neuroprotective effect of hydrogen is limited when used alone since the pathophysiology of post-CA brain injury involves a complex cascade of molecular events (3). A practical solution is to attempt the treatment by targeting a singular altered pathway to combining multi-therapies with mixed effects in conferring neuroprotection and improving survival, such as oxygen therapy, TTM, or pharmacological intervention.

Oxygen plays a pivotal role in post-resuscitation intervention through the compensation of the imbalance between tissue oxygen requirements and supplies. In the current study, the survival rate increased gradually with the increased concentration of oxygen that was mixed with hydrogen. It is worth noting that hydrogen coupled with hyperoxygenation did not aggravate the neuronal damage, but greatly attenuated the oxidative stress. This contradicted the previous animal studies that hyperoxygenation might harm post-ischemic neurons through the production of free radicals and mitochondrial injury (32). Our study was contrary to the previous animal studies that hyperoxygenation might harm post-ischemic neurons through the production of free radicals and mitochondrial injury, because no extra intervention, such as TTM or hydrogen treatment, is given at the same time as oxygen administration in these studies. Indeed, administration of high concentration oxygen during the early post-ischemic reperfusion phase not only increased oxygen delivery to the ischemic tissues but also decreased the oxidative stress-related injuries (24, 33). The increased oxygen extraction together with selectively reduction of the highly toxic hydroxyl radicals and peroxynitrite by hydrogen might contribute to the improved neurological recovery and was the possible molecular mechanism for the neuroprotective effect of hydrogen and high concentrations of oxygen (31).

The neuroprotective effects of hydrogen also have been compared with that of TTM. Using a rat model of VF, Hayashida et al. (10, 13) reported that hydrogen yielded better improvement in neurological recovery and survival to an extent comparable to hypothermia. Using a rat model of asphyxia, we demonstrated that hydrogen inhalation was superior to hypothermia in improving neurological outcomes (14). In the current study, the concomitant administration of hydrogen with hypothermia showed a trend to improve the survival but did not reach statistical significance due to the limited sample size. Additionally, we noticed that there were no significant differences in NDS among groups of animals under hypothermia. This was different from the result that a high concentration of oxygen significantly decreased NDS for animals under normothermia and suggested that hypothermia may facilitate the neurological recovery effect of hydrogen. Therefore, the combination of hydrogen, oxygen, and hypothermia is recommended for post-resuscitation intervention in order to maximize the neurological outcomes.

There are several limitations in the present study. First, the small rodent brain had different metabolic and physiologic properties from the complex human brain. Therefore, the results should be more carefully interpreted when applied to clinical practice. Second, the animal models did not imitate the clinical scenario of CA completely, since the study was performed in healthy animals without underlying cardiac diseases. Third, although we showed that hydrogen inhalation coupled with high concentration oxygen improves survival, the exact mechanism has not been determined. Fourth, all of the interventions performed, including oxygen, hydrogen, and hypothermia, are not brain targeted. Therefore, only studying the neural function without considering the effects of other organs, such as heart, respiration, and kidney, is another limitation of this study.

## Conclusions

In this CA rat model of VF, the neuroprotective effect of hydrogen is independent of TTM but influenced by oxygen concentration. Inhaling hydrogen combined with high concentration oxygen greatly improves survival by reducing oxidative stress, either during normothermia or under hypothermia. The combination of hydrogen with high concentration oxygen and hypothermia is recommended for post-CA care in order to maximize the neurological outcomes.

## Data availability statement

The original contributions presented in the study are included in the article/supplementary material, further inquiries can be directed to the corresponding author.

## Ethics statement

The animal study was reviewed and approved by the Laboratory Animal Welfare and Ethics Committee of the Army Medical University.

## Author contributions

JW and YS contributed to the experimentation, data collection, result analysis, and manuscript composition. JL and BC helped with the experiment and assisted in data collection. YL and CY contributed to the conception and supervision of the research, data interpretation, manuscript drafting, and revision. JW, YS, JL, BC, CY, and YL vouch for the accuracy and completeness of the experiment. All authors have read and approved the submission and publication of the final version of the manuscript.

## Funding

This study was supported by the National Nature Science Foundation of China (NSFC31771070) and the Natural Science Foundation Project of Chongqing (cstc2021jcyj-msxmX0521).

## Conflict of interest

The authors declare that the research was conducted in the absence of any commercial or financial relationships that could be construed as a potential conflict of interest.

## Publisher's note

All claims expressed in this article are solely those of the authors and do not necessarily represent those of their affiliated organizations, or those of the publisher, the editors and the reviewers. Any product that may be evaluated in this article, or claim that may be made by its manufacturer, is not guaranteed or endorsed by the publisher.
